# Mislocalisation of BEST1 in iPSC-derived retinal pigment epithelial cells from a family with autosomal dominant vitreoretinochoroidopathy (ADVIRC)

**DOI:** 10.1038/srep33792

**Published:** 2016-09-22

**Authors:** David A. Carter, Matthew J. K. Smart, William V. G. Letton, Conor M. Ramsden, Britta Nommiste, Li Li Chen, Kate Fynes, Manickam N. Muthiah, Pollyanna Goh, Amelia Lane, Michael B. Powner, Andrew R. Webster, Lyndon da Cruz, Anthony T. Moore, Peter J. Coffey, Amanda-Jayne F. Carr

**Affiliations:** 1 Department of Ocular Biology and Therapeutics, UCL Institute of Ophthalmology, 11-43 Bath Street, London, EC1V 9EL, UK; 2 Moorfields Eye Hospital, 162 City Road, London, EC1V 2PD, UK; 3 Research Department of Haematology, UCL Cancer Institute, 72 Huntley Street, London, WC1E 6DD, UK; 4 UCSF School of Medicine, Beckman Vision Center, 10 Koret Way, San Francisco, CA 94143, USA.

## Abstract

Autosomal dominant vitreoretinochoroidopathy (ADVIRC) is a rare, early-onset retinal dystrophy characterised by distinct bands of circumferential pigmentary degeneration in the peripheral retina and developmental eye defects. ADVIRC is caused by mutations in the *Bestrophin1* (*BEST1*) gene, which encodes a transmembrane protein thought to function as an ion channel in the basolateral membrane of retinal pigment epithelial (RPE) cells. Previous studies suggest that the distinct ADVIRC phenotype results from alternative splicing of *BEST1* pre-mRNA. Here, we have used induced pluripotent stem cell (iPSC) technology to investigate the effects of an ADVIRC associated *BEST1* mutation (c.704T > C, p.V235A) in patient-derived iPSC-RPE. We found no evidence of alternate splicing of the *BEST1* transcript in ADVIRC iPSC-RPE, however in patient-derived iPSC-RPE, BEST1 was expressed at the basolateral membrane and the apical membrane. During human eye development we show that BEST1 is expressed more abundantly in peripheral RPE compared to central RPE and is also expressed in cells of the developing retina. These results suggest that higher levels of mislocalised BEST1 expression in the periphery, from an early developmental stage, could provide a mechanism that leads to the distinct clinical phenotype observed in ADVIRC patients.

Autosomal dominant vitreoretinochoroidopathy (ADVIRC–OMIM 193220) is a rare retinal dystrophy characterised by distinct fundus appearance with a circumferential ring of retinal atrophy and pigmentation in the far peripheral retina^1^,^2^,^3^. The macula is usually normal at diagnosis but cystoid macular oedema and macular atrophy may occur over time^4^. Other ocular features include cataract, angle closure glaucoma, fibrillar condensation of the vitreous, retinovascular abnormalities and developmental abnormalities, such as nanophthalmos and microcornea^5^. The disorder is slowly progressive with a good visual prognosis except in those patients with angle closure glaucoma or retinal neovascularisation. There is wide variability in the phenotype even in members of the same family, the full field ERG may be normal initially but usually deteriorates slowly with increasing age. Most patients show a depressed light peak in the electro-oculogram (EOG)^5^,^6^, indicative of a defect in the retinal pigment epithelial (RPE) cells of the eye.

ADVIRC is by caused mutations in the *Bestrophin 1* (*BEST1*) gene, which encodes a 585 amino acid transmembrane protein expressed in RPE cells^7^. Developmentally, the human *BEST1* promoter is active in RPE cells from early embryonic stages (E9 onwards) in the mouse^8^, and there is evidence to suggest that the promoter is also active in the embryonic neural retina^9^,^10^. Although murine *BEST1* mRNA can be detected in the RPE at E15^11^, BEST1 protein is not detected in the RPE until postnatal day 10, a developmental stage concurrent with the appearance of the ERG a-wave^11^. Interestingly, the distribution of *BEST1* expression varies across the adult eye, with higher levels of expression detected, both at transcript and protein level in peripheral RPE cells compared to macula RPE^12^.

BEST1 is an integral membrane protein that localises to the basolateral membrane of the RPE cell^7^, however, its exact role remains unclear; in overexpression studies BEST1 has been reported to function as a Ca^2+^-activated Cl^−^ channel^13^,^14^,^15^ and a volume-regulated anion channel (VRAC)^16^,^17^. BEST1 has also been shown to influence the kinetics of voltage-gated Ca^2+^ channels^18^,^19^, recruit Ca^2+^ from endoplasmic reticulum (ER) stores^20^, regulate intracellular trafficking^21^ and mediate bicarbonate transport^22^ and neurotransmitter release^23^. Investigations using human induced pluripotent stem cell (iPSC)-derived RPE (iPSC-RPE) from patients with bestrophinopathies suggest that BEST1 influences fluid flux in cells; this may reflect a role for BEST1 as a vital component of the VRAC^16^ or through regulation of ER calcium stores^24^. A recent analysis of BEST1 crystal structure has revealed it to be a 4-transmembrane domain spanning protein which assembles in a pentameric structure to form an calcium-gated anion channel^25^,^26^.

The clinical phenotype of ADVIRC is distinctly different from other bestrophinopathies, such as Best disease (Best vitelliform macular dystrophy), Autosomal Recessive Bestrophinopathy and adult onset vitelliform macular dystrophy, all of which primarily affect the central retina. ADVIRC is a rare disease and until recently only four associated mutations had been identified; p.V86M, p.V239M, p.Y236C^5^ and p.V235A^27^. These mutations do not affect the trafficking of BEST1 to the basolateral membrane of MDCK cells^28^, however they have been shown to alter the splicing of *BEST1* in HEK293 minigene assays, resulting in exon skipping or duplication. These findings lead to the hypothesis that ADVIRC is caused by aberrant *BEST1* pre-mRNA splicing. However, a recent report, which identified and characterised a novel ADVIRC-associated missense mutation, c.248G > A (p.G83D), in the same minigene system, suggests that aberrant splicing may not account for the ADVIRC phenotype^4^. Ideally, the effects of these mutations should be examined in ADVIRC patient RPE cells, however these tissues are rarely available.

iPSC technology offers a new platform to investigate the molecular pathology of eye diseases. iPSCs are produced from somatic cells, such as fibroblasts and blood cells, by overexpressing a small panel of embryonic transcription factors required for pluripotency^29^. Like human embryonic stem cells (HESC), iPSCs have the ability to proliferate indefinitely in an undifferentiated state and are also capable of differentiating into any cell of the body. However, as iPSCs can be reprogrammed from somatic cells taken from any patient, these cells also have the advantage of carrying the genetic background responsible for inherited diseases.

Both HESCs and iPSCs can be easily differentiated into RPE cells by removal of bFGF from the culture medium^30^,^31^, or through directed differentiation protocols, recapitulating the signalling pathways responsible for RPE development *in vivo*^32^,^33^. Pluripotent stem cell derived-RPE (PSC-RPE) cells are readily identified in differentiated stem cell cultures due to their pigmented appearance, which enables the manual removal of pigmented foci for culture as a purified population. The ease of differentiation, identification and purification of RPE from pluripotent stem cell cultures has enabled RPE cells to be fast-tracked into clinical translation as a potential cellular therapeutic for RPE diseases such as age-related macular degeneration and Stargart’s disease^34^ and has established them as a valuable cellular platform in which investigate the molecular mechanisms of bestrophinopathies *in vitro*^24^,^35^.

Here, we have generated iPSC from an ADVIRC family expressing the c.704T > C (p.V235A) mutation, which is thought to result in the duplication of *BEST1* Exon 6. We have differentiated the patient derived iPSCs into RPE, establishing a novel cellular model of ADVIRC, allowing us to investigate the splicing of *BEST1* in a diseased human RPE cell. We found no evidence of alternative splicing of *BEST1* Exon 6 in transcripts generated from patient iPSC-RPE. Immunostaining revealed that BEST1 protein was mislocalised; rather than being targeted specifically to the basolateral membrane, BEST1 was also observed at the apical membrane of patient-derived RPE cells. We have also examined the expression of BEST1 during development in foetal eyes and found that BEST1 expression is highly abundant in peripheral RPE compared to macular RPE, suggesting a possible a mechanism whereby peripheral RPE may be more vulnerable to the affects of mislocalised BEST1 in ADVIRC patients.

## Results

### Pathology of ADVIRC mutation

**Figure 1:**
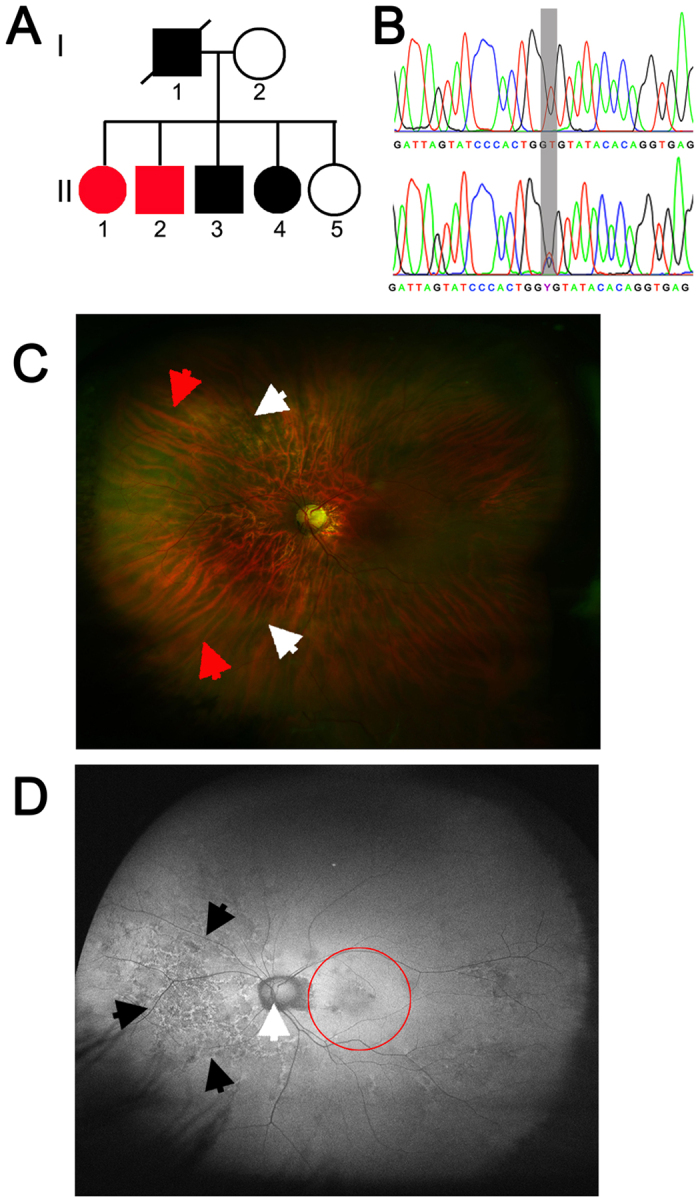
Clinical features of the ADVIRC patient Proband II-2. (**A**) Pedigree of the ADVIRC family investigated in this study. Probands II-1 and II-2, highlighted in red, were used as volunteers for this study. (**B**) Sanger sequencing of control (upper) and proband II-2 (lower) genomic DNA, the grey bar highlights the c.704T > C mutation within Exon 6 in this family. (**C**) Optos widefield colour fundus photograph of the left eye shows the tigroid appearance of the fundus, white arrows highlight the appearance of nasal temporal retinal atrophy, whilst red arrows highlight the circumferential band of pigmentation. The optic nerve is observed as the central yellow structure. (**D**) OPTOS widefield fundus autofluorescence images of the left eye of proband II-2. The black arrows highlight the nasal area of increased autofluorescence signifying RPE cell loss, the white arrow indicates the optic nerve and the red circle indicates the macula area (The subjects eyelashes are observed in the lower portion of the image).

The clinical details of ADVIRC patients, Proband II-1 and II-2 (Fig. 1A), have been described previously^27^. Sequencing confirmed the presence of a c.704T > C missense variation (Rs267606679) in patient cells (Fig. 1B). Proband II-1 was unable to participate in the imaging studies. Optos widefield fundus photography of Proband II-2 shows the tigroid appearance of the extra-foveal retina (Fig. 1C), where a loss of RPE cells allows the underlying choroidal vessels to be observed. Demarcated changes in peripheral retina pigmentation can be observed in a concentric band, alongside nasal and peripapillary area atrophy. OPTOS autofluorescent images show a bilateral pattern of increased mottled autofluorescence that is predominantly nasal, indicative of an RPE cell functional deficit within this area (Fig. 1D and Supplementary Fig. S1A). The peripapillary area surrounding the optic nerve is devoid of RPE cells, whilst the foveal and macular regions appear relatively healthy. Spectral domain optical coherence tomography of the foveal region reveals preservation of the retinal structure in sections of the macula and superior macula (Supplementary Fig. S1B,C) However, Nidek microperimmetry of these regions identifies mild loss of function in the temporal macula and profound loss of function in the superior macula (Supplementary Fig. S1D), suggesting that whilst these central retinal areas appear to be structurally intact there is a deficit in visual function within these discrete regions.

### Generation of ADVIRC patient iPSCs

**Figure 2:**
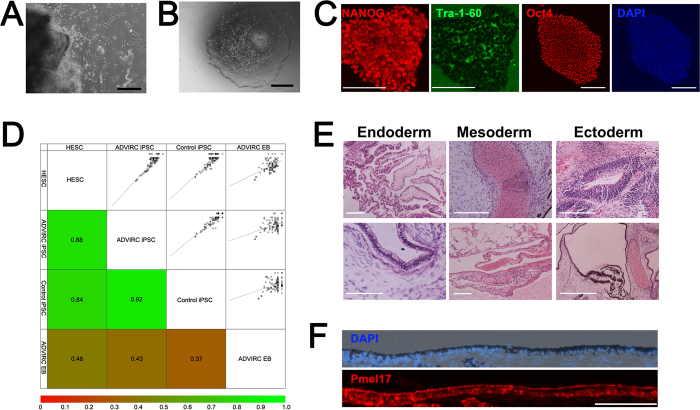
Reprogramming and characterisation of ADVIRC patient-derived iPSCs. (**A**) Growth of fibroblast cells from ADVIRC patient skin explant. Scale Bar 200 μm (**B**) Induced pluripotent stem cell colony reprogrammed from ADVIRC patient. Scale bar 1000 μm (**C**) Immunocytochemistry for stem cell markers confirming pluripotency of ADVIRC patient-derived iPSC. Scale bar for NANOG and TRA-1-60 100 μm, and OCT4 and DAPI 500 μm. (**D**) Taqman^®^ hPSC Scorecard ™ assay results comparing undifferentiated human embryonic stem cells (HESC), undifferentiated control iPSCs (Control iPSC), undifferentiated patient iPSC (ADVIRC iPSC) and differentiated patient iPSC (ADVIRC EB). Scatter plots are shown in the upper right of the matrix and corresponding coefficient of determination (R2) in the lower left. (**E**) Teratoma assay of H&E stained sections taken from ADVIRC patient derived iPSC 8 weeks following injection into NOD-SCID mice displaying tissue from the three germ cell lineages. (**F**) Immunocytochemistry staining of ADVIRC-iPSC derived teratoma sections showing differentiation of pigmented epithelial cells that express the pre-melanosomal protein, Pmel17. Scale bar 100 μm.

A skin punch biopsy was taken from Proband II-1 and II-2 and cultured to permit the expansion of fibroblast cells (Fig. 2A). Patient and control fibroblasts were reprogrammed using episomal vectors. Pluripotent colonies were identified by morphological appearance: individual cells have a high nucleus:cytoplasm ratio, and are densely packed in colonies with defined colony borders that appear phase-positive under the microscope (Fig. 2B). Pluripotency was confirmed by live staining for tumour-related antigen (Tra)-1-60 prior to isolation and expansion of four clonal colonies per patient and control. To verify the undifferentiated state of the patient and control clonal lines, cells were immunostained for the pluripotent stem cell markers, NANOG, OCT4 and TRA-1-60 (Fig. 2C). In order to confirm that patient and control iPSCs are capable of differentiating into cells of the three embryonic germ layers, iPSCs were subjected to pluripotency assays. Quantification of trilineage was performed on pluripotent and differentiated cells using the Taqman hPSC Scorecard™ assay system (Fig. 2D and Supplementary Fig. S2A–C). Comparison of patient and control cells demonstrated that the undifferentiated pluripotent stem cells lines clustered closely to a functionally validated assay reference set (r2 = 0.84–0.92), expressing similar signatures of self-renewal. Self-renewal genes in differentiated iPSCs were down-regulated and markers of ectoderm, endoderm and mesoderm were upregulated, with no lineage bias, implying that the cells have a multilineage differentiation potential. iPSC clonal lines were also subjected to analysis by teratoma assay. iPSCs were implanted into the testis capsule of immune-compromised SCID/NOD mice. After 8 weeks, teratomas were removed (Supplementary Fig. S2D) and subjected to histopathological analysis (Fig. 2E), demonstrating that the iPSCs are capable of differentiating into endoderm (ciliated columnar and cuboidal epithelial cells), mesoderm (hyaline cartilage and adipose tissue) and ectoderm (neural rosettes and pigmented epithelial cells) lineages. The pigmented cells appeared as a component of PAX6 positive optic vesicle-like structures within the teratoma (Supplementary Fig. S3A–C) and formed a distinct polarised PMEL17 positive monolayer, with basal DAPI positive nuclei and apical pigmentation similar to RPE (Fig. 2F). Non-pigmented cells adjoining the PMEL17 positive cells appeared striated and expressed early retinal progenitors markers CRX and RX (Supplementary Fig. S3D,E). All iPSC clonal lines were karyotypically normal (Supplementary Fig. S4).

### Differentiation of iPSC into RPE cells

**Figure 3:**
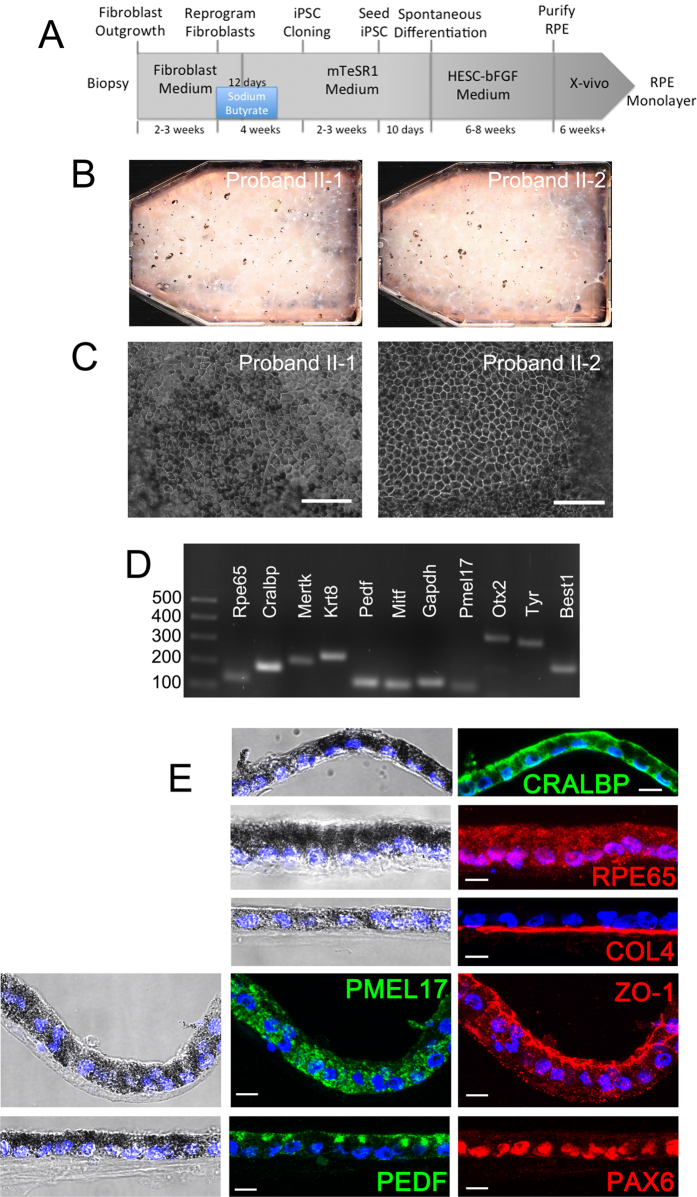
Differentiation and characterisation of ADVIRC patient iPSC RPE. (**A**) Timeline of RPE cell production from initial patient skin biopsy. (**B**) Spontaneous differentiation of RPE cells in ADVIRC patient iPSC cultures 6 weeks following removal of bFGF from the culture medium. (**C**) A monolayer of ADVIRC patient iPSC-RPE after manual purification of pigmented cells from iPSC cultures, scale bar 200 μm (**D**) PCR amplification of RPE cell markers in ADVIRC patient iPSC-RPE cells. (**E**) Immunostaining of ADVIRC patient iPSC-RPE sections. Nomarski and confocal images are merged with DAPI. All scale bars 20 μm.

iPSCs were cultured in mTeSR1 medium until individual stem cell colonies merged, after which the medium was replaced with HESC medium minus bFGF (See Fig. 3A for full timeline of reprogramming and differentiation). Spontaneous differentiation of iPSCs into pigmented RPE cells occurred over a 6–8 week period (Fig. 3B). The pigmented foci were manually dissected, dissociated and re-seeded, forming a pigmented monolayer of epithelial cells, with classic RPE hexagonal cobblestone morphology appearing after 6 weeks (Fig. 3C).

To confirm the identity of the RPE we performed RT-PCR to assess gene expression in iPSC-RPE monolayers (Fig. 3D). iPSC-RPE expressed key markers of RPE cells, including: *MerTK*, *cytokeratin 8* (*KRT8*), *PEDF. pre-melanosome protein 17* (*PMEL17*), *tyrosinase* (*TYR*); *orthodenticle homolog 2* (*OTX2*), *retinal pigment epithelial specific protein 65kDa* (*RPE65*), *cellular retinaldehyde binding protein 1* (*CRALBP*) and *BEST1*.

Vertical sections through patient iPSC-RPE monolayer revealed highly pigmented, polarised cell morphology with a basal nucleus and localisation of pigment granules towards the apical portion of the cells (Fig. 3E). Immunocytochemical staining demonstrated that the cells expressed RPE cell proteins including CRALBP and RPE65. Punctate staining for PMEL17 was observed in the cytoplasm of the cell, PEDF expression was observed at the apical surface and within discrete pockets at the apex of the cell. The expression of apical tight junction marker zona-occludins1 (ZO-1) was localised to the apical portion of the cell, whilst Collagen IV (COL4) was localised to the basement membrane.

### Effects of an ADVIRC mutation on *BEST1* processing in patient iPSC-RPE cells

**Figure 4:**
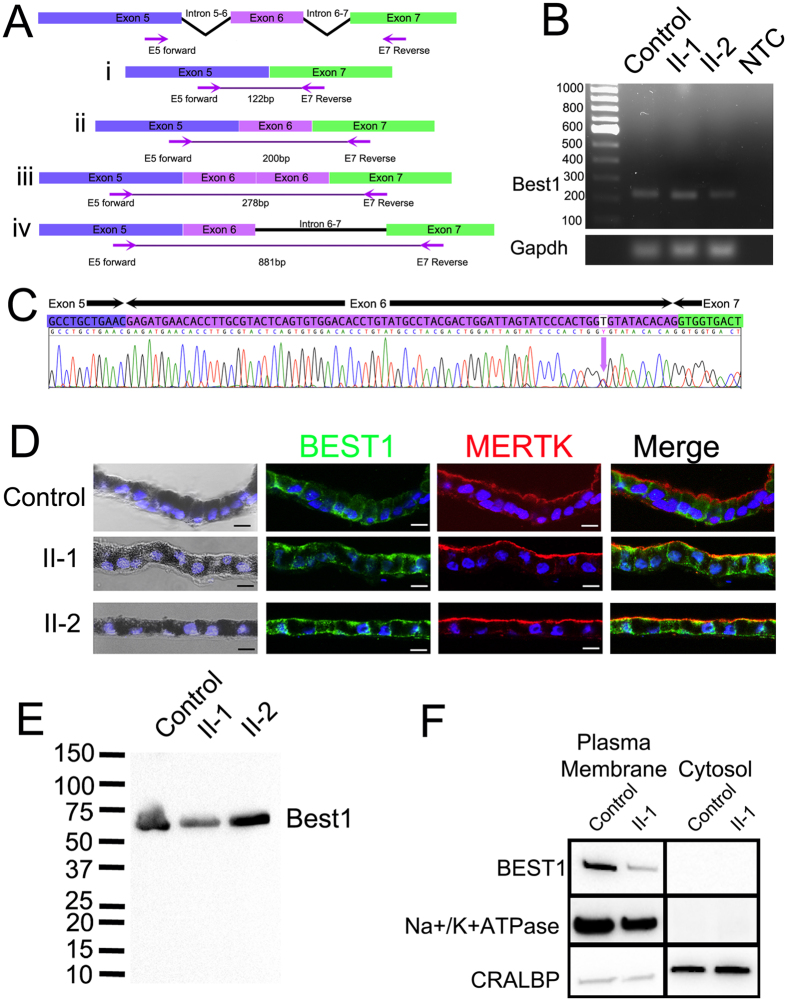
Effects of the pV235A mutation on BEST1 in patient iPSC-RPE cells. (**A**) Representation of possible splicing events caused by the p.V235A mutation and expected amplicon size when amplified with Exon 5–7 spanning cDNA primers i) Exon skipping producing a 122 bp amplicon, ii) normal splicing producing 200 bp amplicon, iii) exon duplication producing a 278 bp amplicon and iv) retention of intron 6–7 producing a 881 bp amplicon (**B**) PCR amplification of cDNA produced from control and patient (Probands II-1 and II-2) iPSC-RPE cells and a non-template cDNA synthesis control (NTC). (**C**) Sequencing of 200 bp amplicon from patient iPSC-RPE reveals normal splicing of exon 6. (**D**) Immunocytochemical detection of BEST1 and MERTK in sections of iPSC-RPE derived from control and ADVIRC patient (II-1 and II-2). The Nomarski images demonstrate the pigmentation of the iPSC-RPE monolayers. DAPI staining of cell nuclei is shown in blue. All scale bars 20 μm. (**E**) Western Blot analysis of BEST1 protein expression in control and patient (II-1 and II-2) derived iPSC-RPE monolayers. (**F**) Subcellular fractionation assay show that BEST1 is detected only in the plasma membrane fractions. Cropped images are displayed, full length blots are included in Supplementary Fig. S6.

A previous study has suggested that the c.704T > C (p.V235A) ADVIRC mutation resides within an enhancer splice site leading to the duplication of Exon 6^27^. We calculated the expected size of splice variants from the *BEST1* transcript (Ensembl ENST00000378043) based on (i) excision of Exon 6, (ii) normal splicing of Exon 6, (iii) duplication of Exon 6 and (iv) retention of the 681bp Intron 6–7 (Fig. 4A). We examined the effects of this mutation on *BEST1* transcripts expressed in iPSC-RPE monolayers. cDNA was prepared from control and patient iPSC-RPE and amplified using primers flanking the intron-exon boundaries of Exon 6 (Fig. 4A). A single amplicon of 200bp was observed in control and patient cells, signifying normal splicing of Exon 6 in ADVIRC patients (Fig. 4B). There was no evidence of any other differentially sized amplicons in the RT-PCR reactions, even after increasing the PCR extension time. Sequencing of the amplicon confirmed its homology to the *BEST1* transcript (NM_004183.3). A single nucleotide mismatch was observed 10 nucleotides from the end of Exon 6, corresponding to the T > C missense variant (Fig. 4C). We also examined products amplified with a 5′Fam-labelled forward primer using DNA fragment analysis by capillary electrophoresis. We detected a highly expressed peak of around 201 bp, a number of smaller peaks between 190–237 bp in all of the iPS-RPE cDNA samples, however, we did not observe a peak at 278 bp, which would correspond to the duplication of the *Best1* Exon 6 (Supplementary Fig. S5A,B). Analysis of iPSC-RPE cell lysates by Western blot produced a band of approximately 68 kDa in control and patient samples, again indicative of normal splicing of *BEST1* in ADVIRC patient RPE cells (Fig. 4E).

### Effects of ADVIRC mutation on BEST1 localisation

In the RPE, BEST1 protein expression is localised to the basolateral membrane^7^. Immunocytochemistry was used to assess the effects of the c.704T > C (pV235A) ADVIRC mutation on BEST1 localisation in patient-derived RPE cells (Fig. 4D). In control cells, BEST1 was observed at the basolateral membrane only, whilst MERTK, a protein required for the phagocytosis of photoreceptor outer segments, was expressed at the apical surface. The localisation of BEST1 differed in patient RPE cells in comparison to controls, rather than being limited to the basolateral membrane, BEST1 staining was also observed at the apical membrane of patient iPSC-RPE. MERTK staining in patient cells was consistent with that of controls, and was observed only at the apical surface of cells, confirming that cells were appropriately polarised and sectioned in the correct orientation. The presence of apical pigmentation, basal nuclei and polarised staining of key RPE markers (MERTK, PEDF, COLIV and ZO-1 from Fig. 3E) suggests that despite the mislocalisation of BEST1, the overall cell polarity is maintained in patient iPSC-RPE. Subcellular fractionation experiments confirmed that BEST1 protein expression was limited to the plasma membrane with no evidence of BEST1 in the cytosolic fraction (Fig. 4F).

### Expression of BEST1 during development of the eye

**Figure 5:**
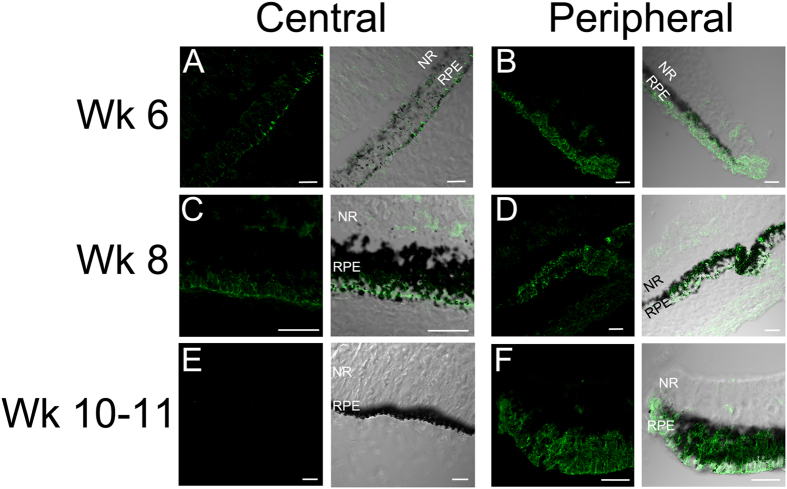
Expression of BEST1 in the developing human eye. BEST1 protein (green) was examined in central (**A,C,E,**) and peripheral (**B,D,F**) regions of the developing human eye at Week 6, 8 and 10–11 post-conception (n = 1). Nomarski images indicate RPE cell pigmentation, the neural retina (NR) and RPE layer are indicated (All scale bars 20 um).

Clinical findings indicate that the initial degeneration in ADVIRC occurs in the periphery, whilst in general, the central macula is relatively intact, structurally and functionally, until later stages^36^. The finding that *BEST1* transcripts and protein are more abundant in the periphery^12^, an area where the initial pigmentary defects are observed in young (<10 years old) ADVIRC patients^2^,^37^, could imply a mechanism by which the increased presence of mislocalised BEST1 in the peripheral area contributes to the distinct ADVIRC pathology and the developmental abnormalities. To investigate whether regional differences exist in the developing eye we examined BEST1 by immunohistochemistry in the human eye during early gestation. At 6 weeks gestation the RPE is evident by the presence of pigmented cells in central and peripheral regions of the eye (Fig. 5). At this stage BEST1 protein is detectable in the basolateral portion of central and peripheral RPE, and appears more abundant in the periphery. Interestingly some immunoreactivity is also observed within cells of the overlying neural retina. A similar pattern for BEST1 immunoreactivity in RPE is observed at Week 8. At Week 10–11 BEST1 immunoreactivity is high within the peripheral RPE, but in this instance, using the same confocal microscope acquisition settings for comparison, we were unable to observe BEST1 immunostaining in the central macular region, suggesting a distinct difference in BEST1 expression levels between these two regions in the developing eye.

## Discussion

The use of iPSCs derived from patients with inherited retinal dystrophies provides an alternative approach to investigate disease mechanisms. Here, we have used iPSC technology to examine the effects of an ADVIRC-associated mutation on *BEST1* expression in patient-derived iPSC-RPE cells. We were able to successfully grow RPE cells from patient and control iPSC cultures. Pigmented foci emerged from iPSC lines of control and patient cells within the same time period and the purified patient and control iPSC-RPE cells were morphologically similar, expressing an array of RPE cell markers that localised to the appropriate cellular domain suggesting that iPSC-RPE were correctly polarised.

Previous studies, relying on HEK 293 cells to examine the effects of ADVIRC-associated mutations, have suggested that ADVIRC mutations affect *BEST1* pre-mRNA splicing^5^,^27^. In this system, the c.704T > C (p.V235A) mutation resulted in the duplication of Exon 6^27^. However, recent investigations into a novel mutation (pG83D) using the same minigene assay in HEK293 cells failed to show any effect on splicing^4^. To test the hypothesis that alterations in splicing cause ADVIRC, we assessed transcript expression in iPSC-RPE cells from patients harbouring the pV235A mutation. We found no evidence of alternative splicing of *BEST1* mRNA in patient cells; the detection of a 200 bp amplicon was indicative of correct *BEST1* splicing.

The alternative splicing of pV235A mutant *BEST1* mRNA observed by Burgess *et al*.^27^ may simply be an artefact of the HEK 293 cell line. HEK293 cells have been widely used as a tool to model recombinant protein expression due to their ease of culture and efficiency of transfection and have been a popular choice to model the effects of *BEST1* mutations on the electrical conductance of cells, despite the incorrect trafficking of wild-type BEST1 to the cytoplasm^38^. The identification of a *BEST1* splice variant in HEK293 cells could highlight a potential difference in gene splicing of a transcript between RPE and non-RPE cells. The correct splicing of pre-mRNA is an essential biological procedure in the processing of eukaryotic transcripts and misregulation of these events can lead to human disease. Splicing mechanisms specific to the RPE cell have been identified previously in mouse models of retinitis pigmentosa, where mutations in ubiquitously expressed precursor mRNA processing factors PRPF3, 8 and 31 (proteins that regulate mRNA splicing) result in degenerative changes specific to the RPE with no syndromic effects detected^39^. This suggests that the correct processing of RPE transcripts may be reliant on an RPE-specific splicing mechanism.

For the first time we have been able to examine the effects of the ADVIRC patient mutation (c.704T > C, p.V235A) on protein localisation in an RPE cell. In this study we have established that BEST1 expression is not limited solely to the RPE basolateral membrane; it is also observed at the apical membrane of patient RPE cells. The RPE is a highly polarised cell that forms a tight barrier between the retina and the underlying choroidal blood supply. The ion transport activities of the RPE are an essential component of maintaining cellular homeostasis as well as controlling ion and fluid composition in the subretinal space. These RPE functions are co-ordinated by a variety of ion channels and receptors that operate in the basolateral and/or apical membranes of the cells. Disruption of this tightly regulated and polarised cell conductance by mislocalisation of BEST1, a proposed ion channel, at the apical and basal surface may lead to the clinical pathology observed in ADVIRC patients.

Overexpression studies in Madin-Darby Canine Kidney (MDCK) cells have been performed previously to assess the effects of *BEST1* mutations on protein localisation in cells. Analysis of various *BEST1* mutations in MDCK cells reveals issues with protein trafficking, resulting in retention of BEST1 in the cytoplasm^21^,^28^,^38^ or displacement to the apical membrane^40^. In contrast to the finding we report here, BEST1 ADVIRC p.V235A variant is reported to correctly localise to the basolateral membrane after overexpression in polarised MDCK cells^28^. This clear discrepancy between protein localisation in iPSC-RPE cells further calls into account the validity of using non-RPE cells to model RPE specific mutations. Asymmetric organisation is a key feature of polarised epithelial cells, and the trafficking of proteins to specific cell compartments allows cells to perform specialised functions. RPE cells are unlike the majority epithelial cells, as the polarity of many proteins is reversed, e.g. MCT1 and Na^+^ K^+^ ATPase are basolateral in MDCK cells, but are apical in RPE^41^. This reversal of trafficking appears to be an adaptive change, rendering the subretinal environment permissive to visual phototransduction^42^. Although common epithelial lines, like MDCK, are good models for understanding epithelial organisation, they may not be a suitable model in which to study the asymmetric sorting of RPE proteins. The ability to differentiate iPSCs into RPE provides a means of investigating human RPE cells carrying a specific disease causing mutation. It would be of great interest to use iPSC-RPE to model other ADVIRC mutations to find out whether mislocalisation of BEST1 to the apical surface of the cell is a common finding in ADVIRC patients. In order to determine whether the clinically distinct ADVIRC phenotype results from the apical mislocalisation of BEST1, it is also imperative that investigations into BEST1 localisation in other bestrophinopathy-related diseases be repeated in a more reliable polarised RPE cell system.

The substitution of a valine at amino acid 235 may interrupt a potential basolateral sorting motif for BEST1, which could account for the incorrect trafficking of BEST1 to the apical membrane. Di-hydrophobic motifs, such as LV (Leucine-Valine), LL, VL and VV, and tyrosine based motifs (YXXΦ), where tyrosine (Y) is followed by two amino acids and a hydrophobic amino acid (Φ), are required for the correct basolateral sorting of a number of proteins in polarised epithelial cells^43^,^44^. The p.V235A ADVIRC mutation is contained within a short amino acid sequence containing two di-hydrophobic motifs (L_234_V_235_Y_236_T_237_Q_238_V_239_V_240_), and interrupts the initial LV motif. Of particular interest is the presence of two other ADVIRC mutations (p.V239M and p.Y236C) within this region, which disrupt a di-hydrophobic VV motif and a tyrosine-based motif (YTQV) respectively. The two remaining known ADVIRC mutations (p.G83D and p.V86M), which although not within di-hydrophobic motifs themselves, reside within a similar amino acid sequence, containing a tyrosine motif bordered by two di-hydrophobic motifs (V_81_L_82_G_83_F_84_Y_85_V_86_T_87_L_88_V_89_V_90_). The clustering of ADVIRC mutations within these discrete regions suggests that a common mechanism for ADVIRC may reside within a specific di-hydrophobic-tyrosine-di-hydrophobic sorting motif, with the apical localisation of BEST1 from the mutated allele accounting for the dominant effect of these mutations. Protein localisation should be investigated further in iPSC-RPE from other ADVIRC patient families to confirm this hypothesis.

The substitution of a valine for an alanine at amino acid 235, may also affect structure of BEST1. Valine and alanine are hydrophobic amino acids, which are commonly found in the interior of proteins due to the presence of hydrophobic side chains. The variability in the number of alkyl groups within the side chain can influence the polarity of the amino acid; valine has the second highest hydropathy index (4.2) of the polar amino acids, whilst alanine has the lowest (1.8)^45^. Although the side chains of both amino acids are relatively non-reactive and rarely participate in protein function, the presence of a valine can restrict protein conformation and may participate in the binding and recognition of hydrophobic ligands^46^. Therefore the p.V235A mutation could affect structural aspects of BEST1 protein. Recent analysis of BEST1 has given new insights into the protein conformation and structure as an ion channel^25^,^26^. Topographically, the p.V235A mutation is situated within the highly conserved cytosolic compartment of the third transmembrane spanning helix (S3b), proximal to the “in” transmembrane domain, and in close proximity to two other known ADVIRC mutations (p.Y236C and p.V293M). The p.G83D and p.V86M mutations are also found proximal to the “in” transmembrane boundary of the second transmembrane spanning region (S2B). Therefore it is possible that ADVIRC mutations within these conserved structural domains also have specific effects on the conformation of BEST1 protein, affecting its functional capacity.

The clinical phenotype and molecular analyses suggest that bestrophinopathies result from primary RPE cell dysfunction. However, ADVIRC is distinct from other bestrophinopathies as the initial dystrophy occurs in the periphery rather than macular area, and is often accompanied by abnormal ocular development, implicating BEST1 in normal eye development. Examining Best1 expression in the embryonic and eye we found that, similar to findings in the adult eye^12^, BEST1 is expressed more abundantly in peripheral RPE compared to the central macula. We have also demonstrated that BEST1 expression is not limited to the RPE, it is also expressed within cells of the neural retina during development. Within the adult eye, BEST1 is observed only in the RPE^7^,^47^, however there is evidence to suggest that the BEST1 promoter is active in Muller cell progenitors during mouse eye development^9^, and that BEST1 protein may also be present in rudimentary photoreceptor outer segments in mouse^11^. Higher levels of BEST1 expression in the periphery during early development may render peripheral RPE more prone to the effects of mutations resulting in the mislocalisation of BEST1 at the apical membrane, and the more widespread expression of BEST1 protein in non-RPE cells could also account for the ocular defects observed in ADVIRC patients. The precise mechanism that leads different *BEST1* mutations to cause both peripheral retina disease (ADVIRC) and maculopathies, such as Best disease, remains to be determined.

Our results suggest that the ADVIRC is not a result of aberrant splicing of BEST1, instead we provide evidence to show that the mislocalised expression of BEST1 may lead to the distinct clinical phenotype observed in these ADVIRC patients.

## Materials and Methods

All animal procedures were performed in accordance with UK Home Office regulations under the Animals (Scientific procedures) Act 1986. The research adhered to the principles of the Declaration of Helsinki. All procedures were reviewed and approved by the Moorfields Eye Hospital Ethics Committee and the Health Research Authority NRES Committee London–Riverside (REC reference 12/LO/0489). Informed consent was obtained from two affected siblings from a British family diagnosed with ADVIRC who kindly donated skin biopsies and blood, and were subjected to OPTOS widefield fundus imaging, autofluorescence spectral domain optical coherence tomography and Nidek microperimetry.

### Derivation of patient fibroblast cells

Following the application of a topical local anaesthetic cream (EMLA–eutectic mixture of local anaesthetics) and administration of a subcutaneous local anaesthetic, a 5 mm punch biopsy was taken from two siblings: Proband II-1, a 63-year-old female and Proband II-2, a 53-year-old male). These subjects have been shown to express a variant heterozygous T > C change at position 704 of *BEST1* (NM_004183.3) coding sequence resulting in the substitution of a valine to an alanine at amino acid 235 (p.V235A) in BEST1 protein^27^.

The patient skin biopsies were collected in warm DMEM medium, dissected into small pieces using a sterile scalpel blade onto 0.2% gelatin-coated tissue plate and cultured in fibroblast medium (DMEM supplemented with 10% Foetal calf serum, 1 x penicillin-streptomycin, 1 x Non Essential Amino Acids, 1 x GlutaMAX. All Thermo Fisher Scientific). The plate was maintained at 37 °C in a humidified incubator with 5% CO_2_ until sufficient fibroblast cell growth had emerged. Fibroblast cells were routinely dissociated from the plate at approximately 80% confluency using TrypLE Select enzyme (Thermo Fisher Scientific) and routinely maintained at a 1:3 split ratio in fibroblast medium.

### Reprogramming of Patient Fibroblast Cells

Cells were reprogrammed using integration free (episomal) vectors. Patient (Proband II-1 and II-2) and control BJ fibroblast cells (Stemgent Inc.) were dissociated in the proliferative phase (<80% confluency) using TrypLE Select, centrifuged and resuspended in 1 x PBS. Cells (1 × 10^6^) were pelleted by centrifugation and resuspended in 100 μl Nucleofector Solution (Lonza) containing 1 μg of each of the following episomal plasmids: pCXLE-hOct3/4-shp53-F, pCXLE-hSK and pCXLE-hUL^48^ (a kind gift from Shinya Yamanaka). Cells were electroporated using a Nucleofector Device (Lonza), resuspended in fibroblast media, plated onto gelatin-coated plates and incubated overnight in a humidified incubator at 37 °C with 5% CO_2_. Culture medium was replaced daily with fibroblast medium containing 0.5 mM Sodium Butyrate for one week. Cells were dissociated and plated onto HESC-qualified matrigel-coated tissue culture plates (Corning) in fibroblast medium containing 0.5 mM sodium butyrate. The following day the medium was changed to mTeSR1 medium (STEMCELL Technologies) containing 0.5 mM Sodium Butyrate. The medium was replaced daily for four days, after which it was replaced daily with mTeSR1 medium alone until iPSC colonies appeared. Reprogrammed colonies were identified by morphology, and pluripotency confirmed using StainAlive DyLight 488 antibodies to human TRA-1-60 and Tra-1-81 (Stemgent Inc.). Clonal iPSC colonies were isolated by manual dissection using a sterile Pasteur pipette, transferred to HESC-qualified Matrigel-coated culture dishes and cultured in mTeSR1 medium. Cell medium was replaced daily and cells passaged by manual dissection approximately every 5 days to maintain pluripotency. Unless stated, data shown in the results are from Proband II-2-derived cells.

### Scorecard Analysis

Patient-derived and control iPSCs were analysed for pluripotency using the Taqman hPSC Scorecard Assay (Thermo Fisher Scientific). Undifferentiated cells and randomly differentiated cells through embryoid (EB) formation were prepared for the plate according to the manufacturer’s instructions. Cells were collected in TRIzol reagent on Day 8. RNA was extracted, and cDNA prepared from iPSC and differentiated cells according to the manufacturer’s protocol for hPSC Scorecard analysis. The qRT-PCR was performed on a StepOne Real-Time PCR system using the manufacturer’s ScoreCard experimental template file. Gene expression data was assessed using hPSC Scorecard Analysis Software (Thermo Fisher Scientific).

### Teratoma formation

iPSCs were cultured to 60–70% confluency on HESC-qualified matrigel-coated plates in mTeSR1 medium. Cells were dissociated using dispase and resuspended in 30% Matrigel and 70% mTeSR1. The cell suspension was injected into the testes capsule of 6–8 week-old NOD-SCID mice under inhaled isofluorane anaesthesia. Animals were sacrificed by carbon dioxide asphyxiation after 8 weeks, and teratomas excised and fixed in Histochoice (Amresco). Teratomas were dehydrated in increasing alcohol solutions, cleared using xylene and embedded in paraffin wax. The tissue was sectioned onto charged glass slides (VWR), de-paraffinised in xylene and rehydrated using decreasing alcohol solutions and stained with hematoxylin and eosin. Sections were then dehydrated, cleared and mounted.

### Cytogenetic Analysis of iPSCs

iPSCs were cultured to approximately 60% confluency on matrigel coated flasks in mTeSR1 medium and processed for G-banding analysis by TDL Genetics, London.

### Differentiation of cells into RPE

iPSCs were passaged by manual dissection of colonies and re-plated onto flasks coated with Growth Factor-reduced Matrigel and cultured in mTeSR1 medium. The cell culture medium was replaced daily until individual iPSC colonies merged and became confluent. At this point mTeSR1 replaced with HESC media-bFGF (80% DMEM, 20% Knockout serum replacement, 1% Non-essential amino acids, 1 mM L glutamine, 0.1 mM β-mercaptoethanol, 30 μg/ml gentamicin) and the cell medium replaced twice weekly. Cells were allowed to spontaneous differentiate for approximately 6–8 weeks, at which point distinct pigmented colonies could be observed. The pigmented foci were dissected manually using a crescent blade knife (Interfocus). The foci were dissociated by incubation in Accutase cell dissociation solution (Sigma-Aldrich Co.) at 37 °C for 2–3 hours; remaining cell aggregates were removed using a 40 μm cell strainer. Purified pigmented cells were plated at a density of 50,000 cells/cm^2^ onto Growth Factor-reduced matrigel-coated plates in X-vivo media (Lonza). The medium was replaced twice weekly for approximately 6 weeks, until a confluent pigmented monolayer had formed. For comparative analysis of the iPSC-RPE monolayers, all cells were cultured for the same amount of time following differentiation.

### Confirmation of patient mutation

Genomic DNA was extracted from fibroblast cells using the GeneElute Mammalian Genomic DNA mini prep kit (Sigma-Aldrich Co). Coding sequences of *BEST1* (Exons 2–11) were amplified using Exon spanning primers (Primer sequences for amplification of Exons 5–6 can be found in Supplementary Table S1) with GoTaq Green Master Mix in a Veriti Thermal cycler. PCR products were separated on an agarose gel, and amplicons extracted and purified using the QIAquick Gel Extraction Kit (Qiagen). Cycle sequencing was performed on each exon specific amplicon using the BigDye Terminator v3.1 sequencing kit (Thermo Fisher Scientific) according to the manufacturer’s protocol. Extension products were purified using Ethanol/EDTA/sodium acetate precipitation. Sample capillary electrophoresis was performed using a 3730 DNA Analyser. Sequences were evaluated in MacVector v14.0.4 (MacVector Inc.) and compared to *BEST1* genomic sequence (NG_009033.1).

### Immunostaining

iPSC and iPSC-RPE cells were fixed with 4% paraformaldehyde at 4 °C for 30 min and washed in PBS. For RPE sections, a monolayer of iPSC-RPE cells was carefully removed from the tissue culture dish, post-fixation, using a cell scraper and transferred to a bijou tube containing 30% sucrose for cryopreservation overnight at 4 °C. Monolayers of cells were embedded at a vertical on-edge position in OCT compound and frozen in a dry ice/acetone bath. Blocks were maintained at 80 °C prior to sectioning at 14 μm onto charged slides using a Leica CM1850 cryostat. Embryonic tissue, obtained from the Human Developmental Biology Resource, was fixed, cryopreserved, embedded and sectioned as above.

Cells/sections were permeabilised by incubation in 0.3% Triton for 10 min and incubated in blocking solution (3% bovine serum albumin and 5% normal donkey serum in PBS) for 1 hour. Cells/sections were incubated for 3 hours at 4 °C in blocking solution containing primary antibodies raised in mouse: TRA-1-81 (1:500, Thermo Fisher Scientific), PMEL17 (1:500, Dako UK Ltd.), PEDF (1:1000, Millipore), CRALBP (1:100, Thermo Fisher Scientific), BEST1 (1:1000, Abcam), and in rabbit: OCT4 (1:1000, Abcam), NANOG (1:1000, Abcam), MERTK (1:500, Abcam) ZO-1 (1:500, Zymed), PAX6 (1:300, Covance), RPE65 (1:200, a kind gift from Dr T. Michael Redmond, National Eye Institute, MD, USA) and COLIV (1:100 Bio-Rad). Cells were washed with PBS and incubated with Alexa Fluor^®^ 555 or 488 conjugated IgG secondary antibodies (1:500, Abcam) in blocking solution for 2 hours. Cells were washed with PBS and mounted using Vectashield Antifade mounting medium with DAPI (Vector Labs). Cells were imaged using a Zeiss LSM 700 confocal microscope and images captured using ZEN software.

### Western Blot

Cell lysates were prepared from samples as previously described^49^. Equal amounts of protein were separated on a Mini-PROTEAN TGX gel and transferred onto PVDF membrane using the Tran-Blot Turbo Transfer System (all Bio-Rad). Membranes were blocked in 10% BSA in PBS-0.05% Tween (PBS-T) for 2 hours and incubated overnight with BEST1 primary antibody raised in mouse (Abcam). Membranes were washed in PBS-T and incubated with polyclonal goat anti-rabbit HRP-conjugated secondary antibody (1:2,000, Dako UK Ltd.) 10% BSA in PBS-T for 2 hours. Membranes were then washed in PBS-T and incubated in Clarity Western ECL Substrate. Bands were detected in a ChemiDoc™ imaging system and analysed with Image Lab software (Bio-Rad).

### Subcellular fractionation

iPS-RPE cells were collected in subcellular fractionation buffer (250 mM sucrose, 20 mM HEPES, 10 mM KCl, 1.5 mM MgCl_2_, 1 mM EDTA, 1 mM EGTA, 1 mM PMSF, 10 ng/ml leupeptin, 1 mM DTT, 50 ng/ml aprotinin, 10 mM NaF, and 100 μM sodium vanadate). Lysates were passed through a 25 Ga needle and incubated on a tube rotator for 30 min at 4 °C. The mitochondrial and nuclear fractions were collected by centrifugation at 10, 000 × g for 5 min at 4 °C. The supernatant was subjected to ultracentrifugation at 100,000 × g for 1 hour at 4 °C. The supernatant, containing the cytosolic fraction was collected. The pelleted membrane fraction was then washed with subcellular fractionation buffer, centrifuged for 45 mins and the pellet resuspended in subcellular fractionation buffer. The samples were mixed with Laemmli buffer and analysed by Western Blot, as described above, using primary antibodies for BEST1 (Abcam, mouse, 1:1000), CRALBP (Thermo Fisher Scientific, 1:1000) and Na^+^/K^+^ ATPase (Abcam, mouse, 1:1000).

### PCR amplification of RPE specific genes

RNA was extracted from patient iPSC-RPE monolayers using TRIzol reagent. The RNA was DNase-treated and used as a template for cDNA synthesis with the SuperScript III First-Strand Synthesis System (Thermo Fisher Scientific) as previously described^31^. PCR was performed on the cDNA in a Verity Thermal Cycler (Thermo Fisher Scientific) using gene-specific primers with Go Taq DNA Polymerase (Promega) according to the manufacturer’s instructions. Products were resolved on a 1.5% agarose gel alongside a 100 bp DNA ladder. Gene specific primer sequences for Otx2 can be found in Supplementary Table S1, gene specific primers for all other genes have been published previously: *Rpe65*, *Cralbp1*, *Pedf*, and *Mertk*^50^; *Krt8*, *Gapdh* and *Tyr*^51^; and *Mitf*, *Pmel17*, *Tyr* and *BEST1*^31^.

### Analysis of Exon 6 Splicing

Primers were designed to amplify a region of the *BEST1* cDNA spanning the exon boundaries of Exon 6 (nucleotides 1217–1294 of the *BEST1* transcript NM_004183.3, Primer sequences for Exon 5–7 spanning primers can be found in Supplementary Table S1). Primers amplifying nucleotides 1153–1352 of *BEST1* were used in a PCR reaction with cDNA synthesised from patient and control iPSC-RPE. In order to amplify possible splice variants, the PCR reaction was performed with extension times of 30–90 seconds. PCR reaction products were separated on a 1.5% agarose gel and prepared for terminator dye sequencing with the E5 Forward or E7 reverse primers as described above. For DNA fragment analysis by capillary electrophoresis, the Exon 5–7 spanning cDNA forward primer (Table S1) was labelled at the 5′-end with FAM (Eurofins Genomics) and PCR reactions prepared as above. Following amplification, 1 μl of reaction product was added to 10 μl Hi-Di formamide containing GeneScan 500 ROX™ dye Size Standard (1:50 dilution), the mix was heated at 95 °C for 5 min and then placed on ice. PCR fragments were separated by size using capillary electrophoresis on a 3730 DNA analyser (Thermo Fisher Scientific), and the relative size of each fragment determined using GeneMarker software (SoftGenetics).

## Additional Information

**How to cite this article**: Carter, D. A. *et al*. Mislocalisation of BEST1 in iPSC-derived retinal pigment epithelial cells from a family with autosomal dominant vitreoretinochoroidopathy (ADVIRC). *Sci. Rep.* **6**, 33792; doi: 10.1038/srep33792 (2016).

## Supplementary Material

Supplementary Information
