# Supplementary material for: Mislocalisation of BEST1 in iPSC-derived retinal pigment epithelial cells from a family with autosomal dominant vitreoretinochoroidopathy (ADVIRC)
Source: Sci Rep. 2016 Sep 22;6:33792. doi: 10.1038/srep33792 (PMC5031956; doi:10.1038/srep33792)
Supplement: Supplementary Information [file srep33792-s1.pdf]

Supplementary Information

Title

Mislocalisation of BEST1 in iPSC-derived retinal pigment epithelial cells from a patient with autosomal dominant vitreoretinochoroidopathy (ADVIRC)

Author List

David A. Carter, Matthew J.K.Smart, William V.G Letton, Conor M. Ramsden, Britta Nommiste, Li Li Chen, Kate Fynes, Manickam N. Muthiah, Pollyanna Goh, Amelia Lane, Michael B. Powner, Andrew R. Webster, Lyndon da Cruz, Anthony T. Moore, Peter J. Coffey, Amanda-Jayne F. Carr.

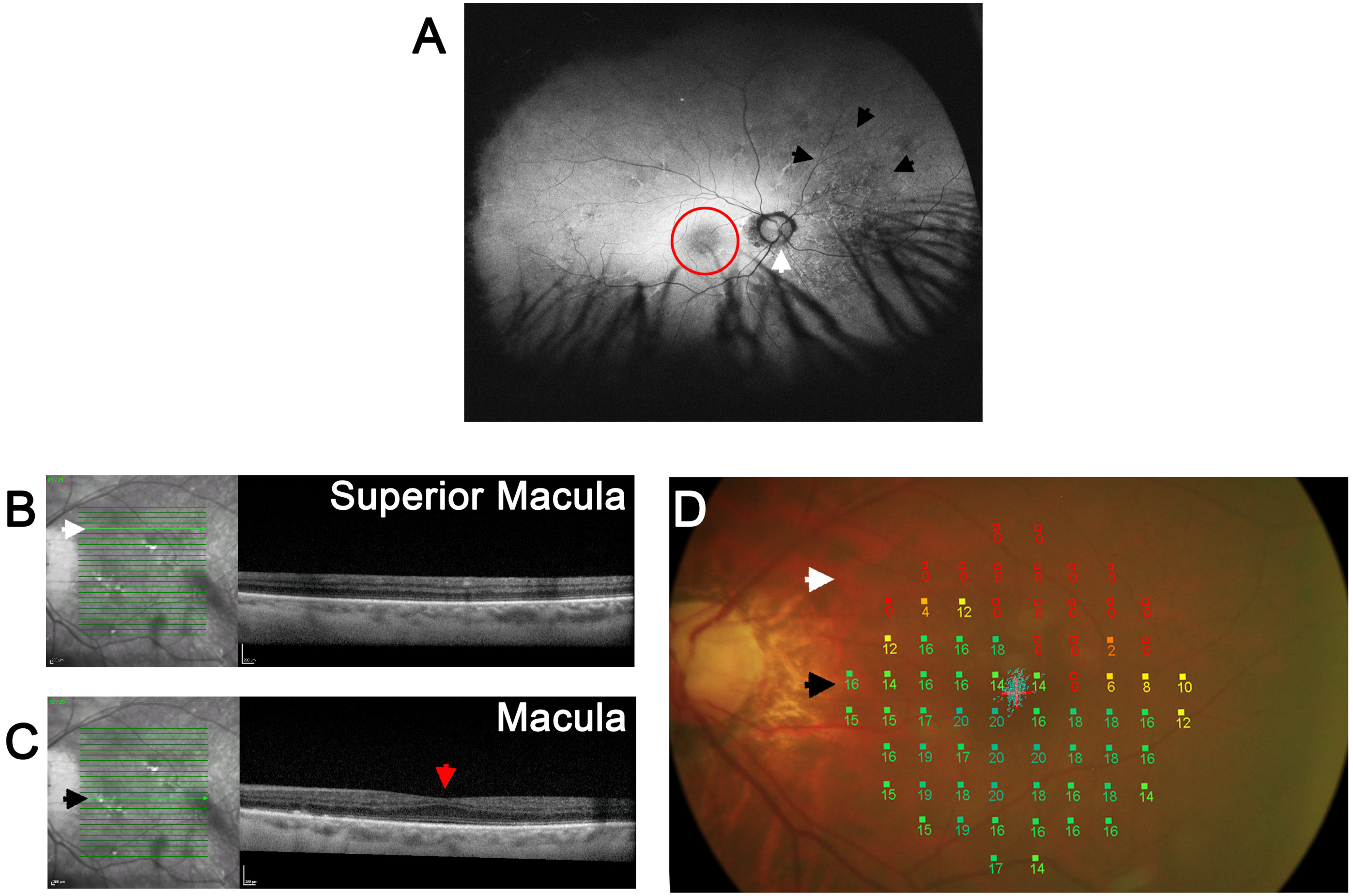

Supplementary Figure 1. Clinical features of the ADVIRC patient Proband II-2 (A) OPTOS widefield fundus autofluorescence images of the right eye demonstrating the bilateral features of ADVIRC. Black arrows highlight the nasal area of increased autofluorescence signifying RPE cell loss, the white arrow indicates the optic nerve and the red circle indicates the macula area (The subjects eyelashes are observed in the lower portion of the image). Spectral domain optical coherence tomography of the left eye, sections of the (B) superior macula and (C) macula indicate preservation of the retinal structure including the photoreceptor cell layers. The foveal pit is indicated by the red arrow. White and black arrows indicate the sampling line of the superior macula and macula region respectively (**D**) Nidek microperimetry of the left eye reveals mild loss of function across the central macula (black arrow) and more profoundly in the superior macula (white arrow). Visual sensory outcomes are graded on an attenuation scale of 0 dB (red – lowest sensitivity) to 20 dB (green – highest sensitivity). Fixation patterns are indicated by the blue spots, with average fixation indicated by the red cross. Black and white arrows indicate equivalent lines examined by OCT.

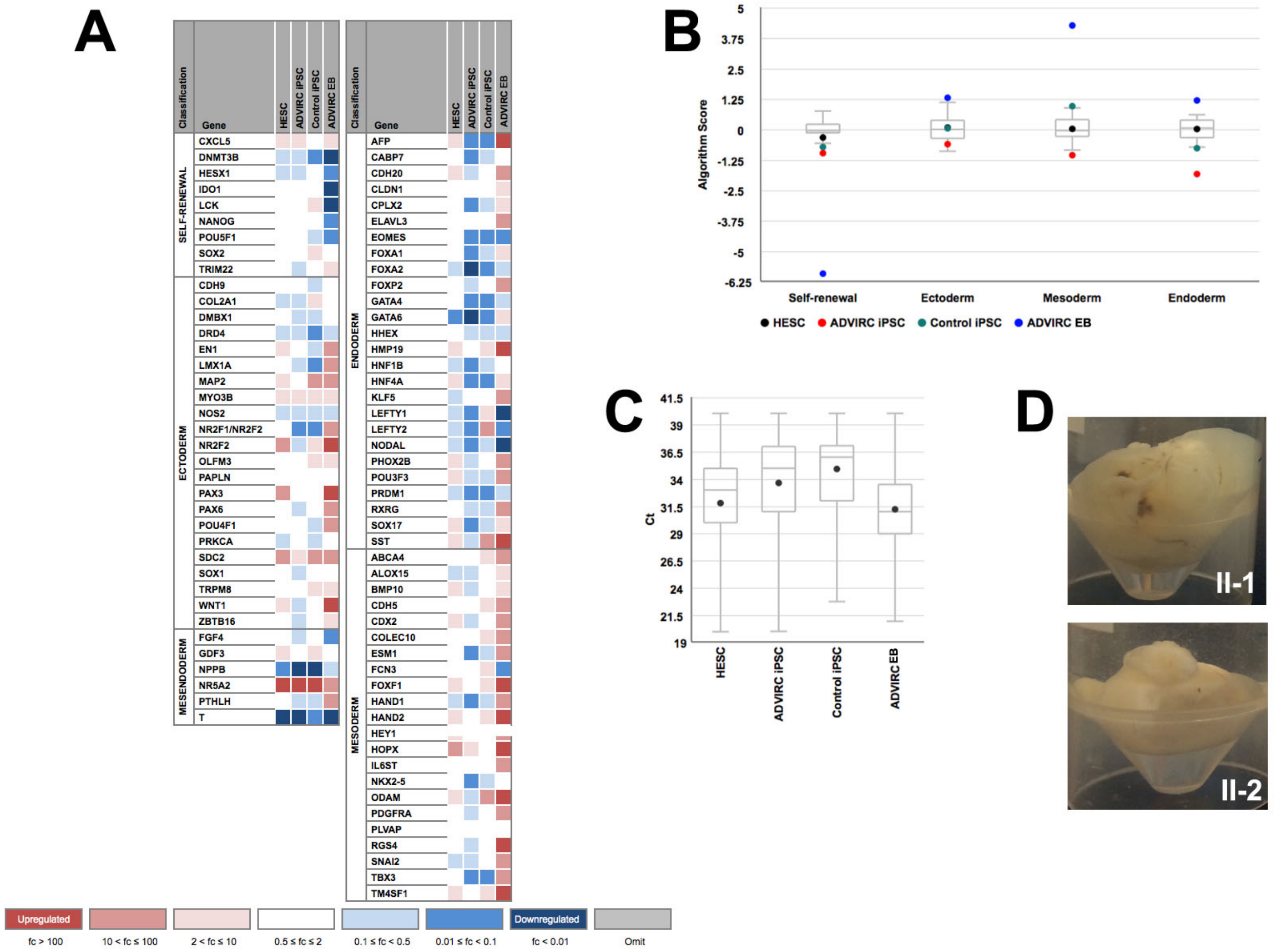

Supplementary Figure 2. Confirmation of iPSC cell pluripotency (A-C) Taqman® hPSC Scorecard ™ assay analysis of pluripotency comparing HESC, Control iPSC, ADVIRC proband II-2-derived iPSC (ADVIRC iPSC) and embryoid bodies derived from ADVIRC proband II-2 iPSC cells (ADVIRC EB) (**A**) Heatmap showing gene expression relative to the Scorecard™ pluripotency gene reference set. (**B**) Assay quality control showing range of CT values for the 96 Scorecard genes. (**C**) Scorebox and whisker plot showing scores of each sample in relation to the Scorecard ™ reference set (represented by the grey box). (**D**) Teratoma formation 8 weeks following injection of iPSC derived from proband II-1 and II-2 into the testes capsule of NOD/SCID mice.

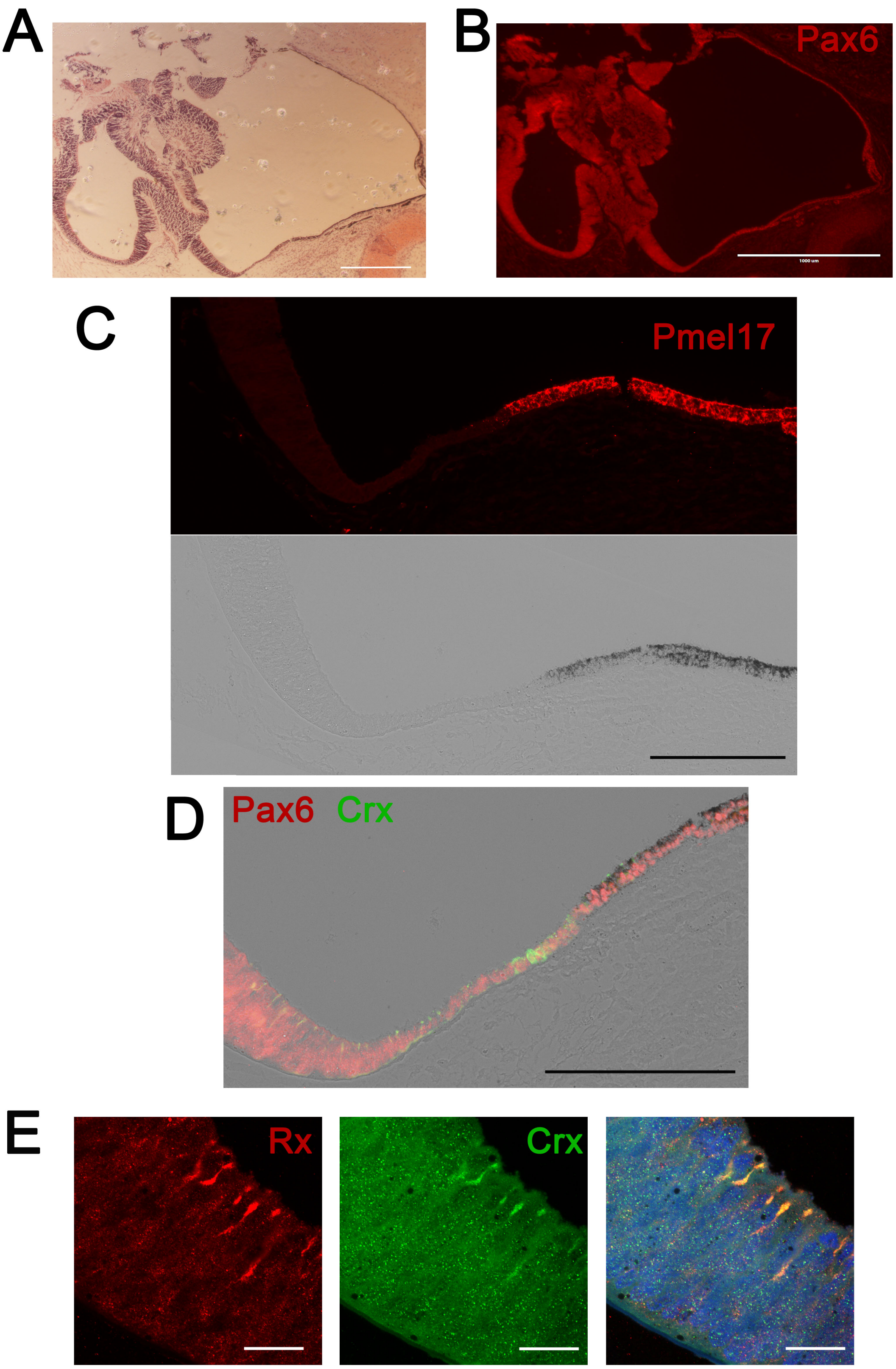

Supplementary Figure 3. Development of optic vesicle-like structures in patient-derived iPSC teratomas. (A) H and E staining of optic vesicle-like structure within ProBand II-2 iPSC teratoma. Scale bar 500µm (B) Expression of Pax6 throughout the optic vesicle-like structure. Scale bar 1000µm (C) Expression of Pmel17 is limited to pigmented cells within the optic vesicle-like structures. Nomarski image shows pigmented cells with DAPI nuclear staining (blue). Scale bar 100µm (D) Pax6 is expressed in all cells of the optic-vesicle like structure, whilst Crx is observed in non-pigmented cells. Fluorescent image is overlaid on Nomarski image to illustrate cell pigmentation. Scale bar 200µm. (E) Expression of the neural retinal cell markers, Crx and Rx, in the striated portion of the optic vesicle-like structure, Scale bars 20µm.

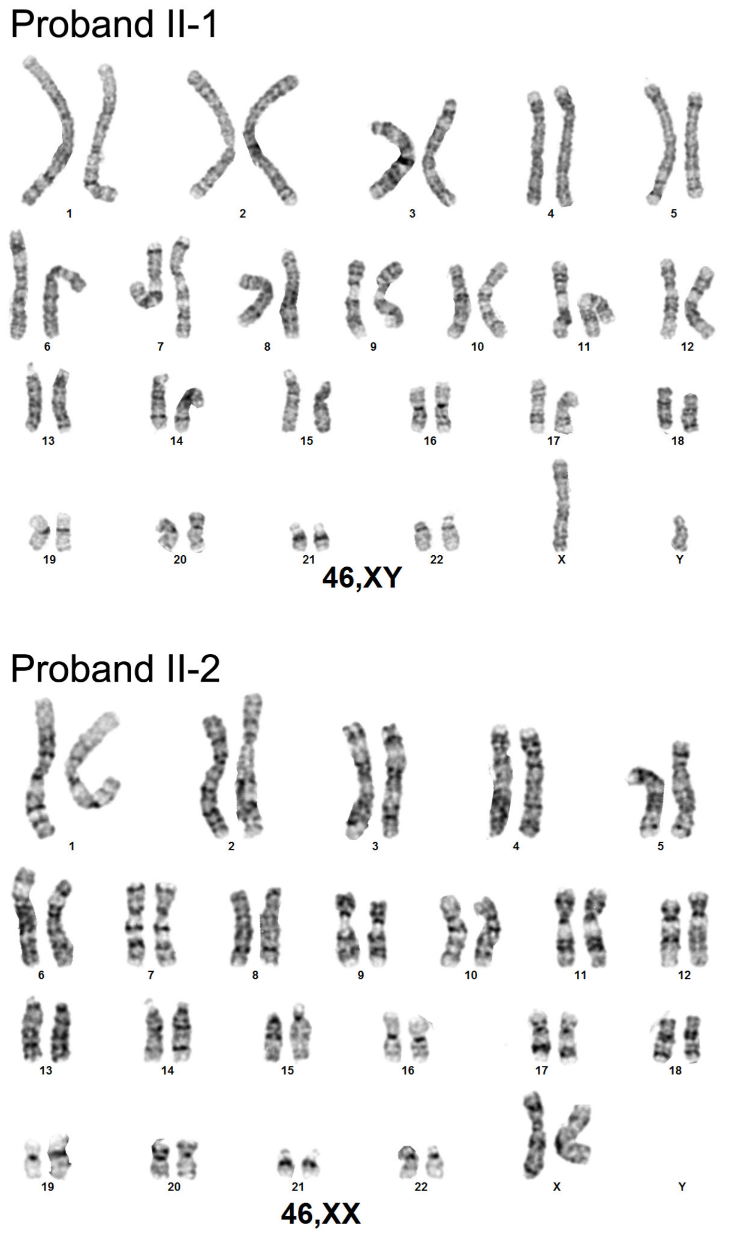

Supplementary Figure 4. Karyotype analysis of ADVIRC iPSCs. G-banded karyotype of ADVIRC patient derived iPSCs from Proband II-1 and II-2.

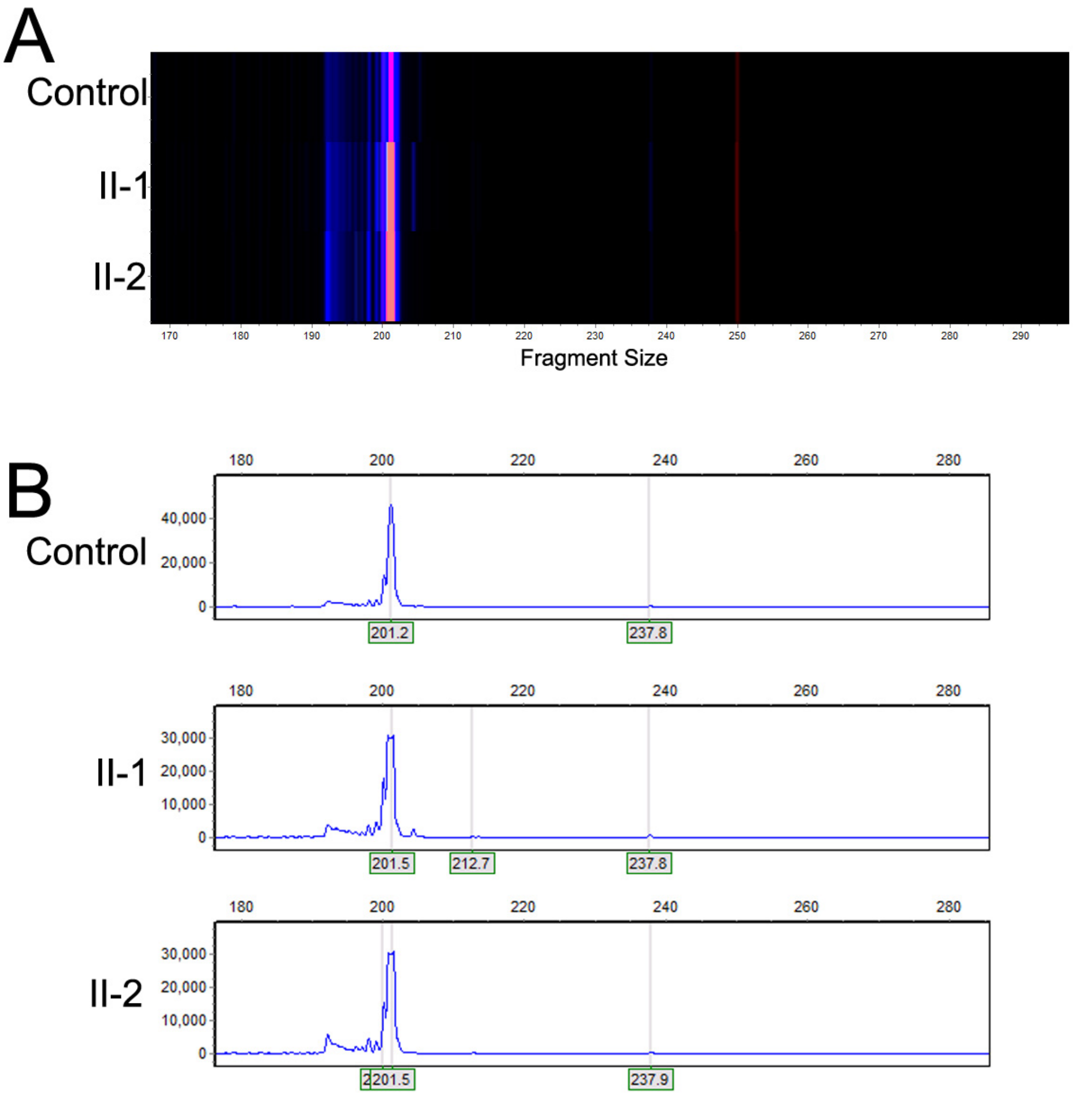

Supplementary Figure 5. DNA fragment analysis of *BEST1* transcripts in ADVIRC patient iPSC-RPE cells. (A) Capillary electrophoresis array view of the PCR fragments generated from control and patient iPSC-RPE. amplified using a 5'Fam-labelled oligonucleotide in (B) Plots of sample peak fragments. A highly expressed peak of approx. 201 bp was observed in all iPS-RPE cells. Other were observed between 190-200bp and at 238bp. No peak was detected at 278bp in any of the samples.

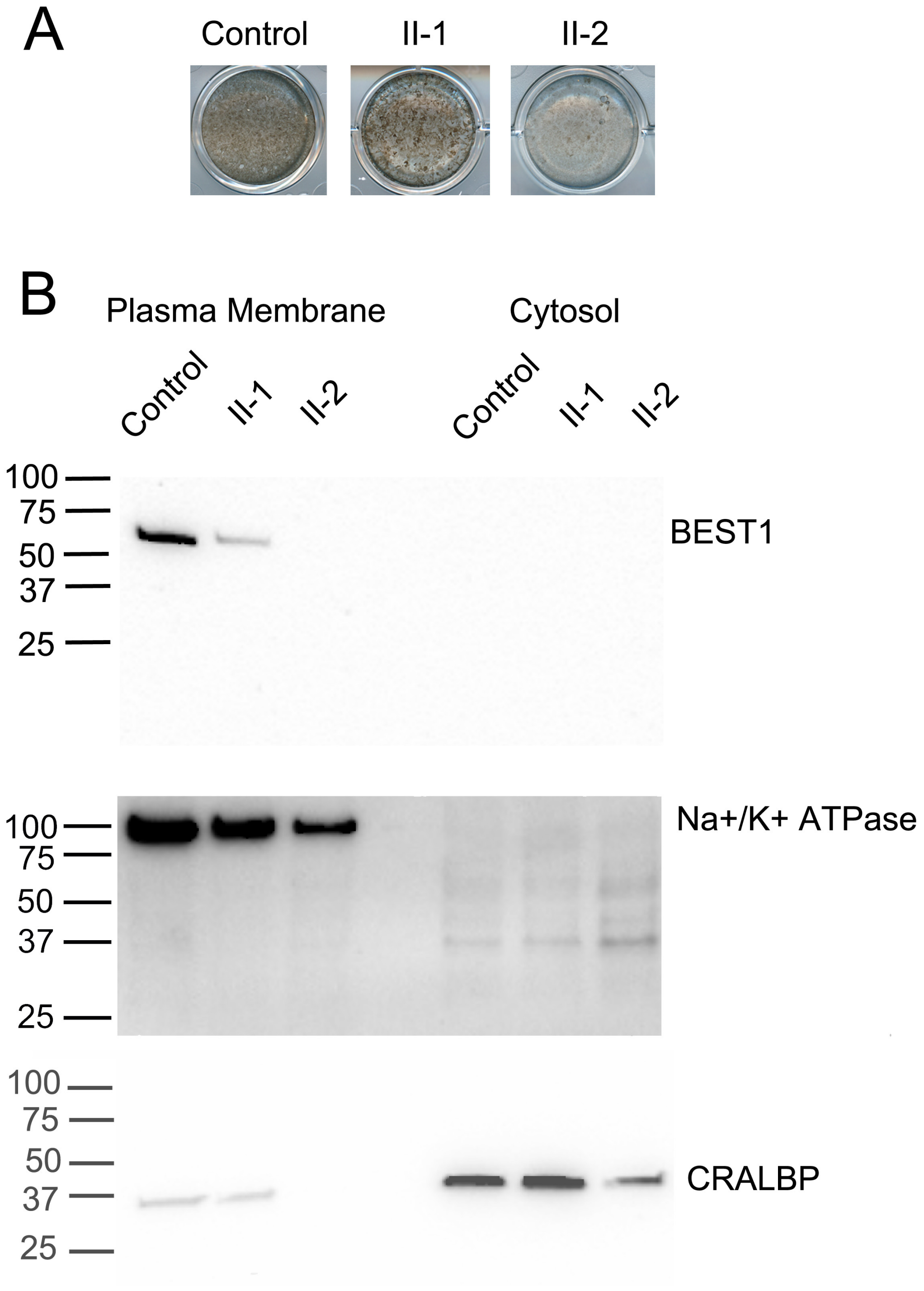

Supplementary Figure 6. Subceullular fractionation assay blots (A) RPE cultures used for the subcellular fractionation assay (B) Individual blots examining BEST1, Na+/K+ ATPase and CRALBP in iPSC-RPE plasma membrane and cytosol fractions.

Supplementary Table 1 Primer Sequences

Target	Forward Primer (5'-3')	Reverse Primer (5'-3')	Tm-3 (^{O}C)	Amplicon size (bp)	Sequence ID
Otx2	GCGCAGCTAG ATGTGCTGGA	CACTGCTGCTG GCAATGGTC	58	297	NM_021728.3
Exon 5-6 sequencing primer	CTTCCATTCCT GCCGCGCCCA TCTC	CATCCCTTCTG CAGGTTCTC	56	729	NG_009033.1
Exon 5-7 spanning CDNA primers	GTCAATGAAG GCGTGGCTTG	GAAACTGCCGC CCAACTAGA	56	200	NM_004183.3
